# *Vital Signs:* Pharmacy-Based Naloxone Dispensing — United States, 2012–2018

**DOI:** 10.15585/mmwr.mm6831e1

**Published:** 2019-08-09

**Authors:** Gery P. Guy, Tamara M. Haegerich, Mary E. Evans, Jan L. Losby, Randall Young, Christopher M. Jones

**Affiliations:** ^1^Division of Unintentional Injury Prevention, National Center for Injury Prevention and Control, CDC; ^2^Division of Toxicology and Human Health Sciences, Agency for Toxic Substances and Disease Registry; ^3^National Center for Injury Prevention and Control, CDC.

## Abstract

**Background:**

The CDC Guideline for Prescribing Opioids for Chronic Pain recommends considering prescribing naloxone when factors that increase risk for overdose are present (e.g., history of overdose or substance use disorder, opioid dosages ≥50 morphine milligram equivalents per day [high-dose], and concurrent use of benzodiazepines). In light of the high numbers of drug overdose deaths involving opioids, 36% of which in 2017 involved prescription opioids, improving access to naloxone is a public health priority. CDC examined trends and characteristics of naloxone dispensing from retail pharmacies at the national and county levels in the United States.

**Methods:**

CDC analyzed 2012–2018 retail pharmacy data from IQVIA, a health care, data science, and technology company, to assess U.S. naloxone dispensing by U.S. Census region, urban/rural status, prescriber specialty, and recipient characteristics, including age group, sex, out-of-pocket costs, and method of payment. Factors associated with naloxone dispensing at the county level also were examined.

**Results:**

The number of naloxone prescriptions dispensed from retail pharmacies increased substantially from 2012 to 2018, including a 106% increase from 2017 to 2018 alone. Nationally, in 2018, one naloxone prescription was dispensed for every 69 high-dose opioid prescriptions. Substantial regional variation in naloxone dispensing was found, including a twenty-fivefold variation across counties, with lowest rates in the most rural counties. A wide variation was also noted by prescriber specialty. Compared with naloxone prescriptions paid for with Medicaid and commercial insurance, a larger percentage of prescriptions paid for with Medicare required out-of-pocket costs.

**Conclusion:**

Despite substantial increases in naloxone dispensing, the rate of naloxone prescriptions dispensed per high-dose opioid prescription remains low, and overall naloxone dispensing varies substantially across the country. Naloxone distribution is an important component of the public health response to the opioid overdose epidemic. Health care providers can prescribe or dispense naloxone when overdose risk factors are present and counsel patients on how to use it. Efforts to improve naloxone access and distribution work most effectively with efforts to improve opioid prescribing, implement other harm-reduction strategies, promote linkage to medications for opioid use disorder treatment, and enhance public health and public safety partnerships.

*On August 6, 2019, this report was posted online as an *MMWR *Early Release.*

## Introduction

Among the 70,237 drug overdose deaths in the United States in 2017 (the last year for which complete data are available), a total of 47,600 (67.8%) involved opioids (*1*). Millions of Americans are at increased risk for an opioid overdose, including persons who use illicit opioids, those who use or misuse prescription opioids, and those with an opioid use disorder (*2*). A population particularly at risk includes persons who use illicit drugs (e.g., cocaine and methamphetamine) that might be mixed with illicit opioids (*3*). The CDC Guideline for Prescribing Opioids for Chronic Pain recommends considering prescribing naloxone when factors that increase risk for overdose are present (e.g., history of overdose or substance use disorder, opioid dosages ≥50 morphine milligram equivalents [MME] per day [high-dose], and concurrent use of benzodiazepines) (*4*). Given that approximately two thirds of overdose deaths involved opioids, 36% of which in 2017 were prescription opioids (*1*), the distribution of naloxone to reverse an overdose is an important element of the public health response to the opioid overdose epidemic (*5*).

For decades, emergency medical service (EMS) providers, first responders, and emergency department clinicians have administered naloxone in cases of suspected drug overdose, and community-based organizations have offered naloxone through education and distribution programs. Recent efforts have focused on expanding naloxone access through clinician prescribing and pharmacy dispensing. All 50 states and the District of Columbia have enacted laws permitting pharmacy-based naloxone dispensing (*6*). Laws allowing providers to prescribe naloxone to any persons in a position to assist another with an overdose (i.e., third-party prescriptions) and standing orders for pharmacists to dispense naloxone have been associated with increases in naloxone dispensing from retail pharmacies (*7*). Several states have mandated that clinicians coprescribe naloxone when overdose risk factors (e.g., high opioid dosages) are present, a recommendation for consideration in the CDC Guideline for Prescribing Opioids for Chronic Pain (*4*); such laws have been associated with substantial increases in naloxone dispensing (*8*). Many of these states have only recently implemented these laws; thus, sufficient time has not passed to examine their full impact at the state or county level.

Recent analyses examining the extent and characteristics of pharmacy-based naloxone dispensing are lacking. Also unknown is the extent to which naloxone dispensing varies by county and by other factors (e.g., prescriber specialty and patient insurance coverage). Understanding variation could help identify the need for tailored approaches to improve prescribing and dispensing, similar to those that have been indicated for opioid prescribing (*9*). To address this gap and to inform future overdose prevention and response efforts, CDC examined trends in, and characteristics of, naloxone dispensing from retail pharmacies at the national and county levels in the United States.

## Methods

Data on naloxone dispensing came from IQVIA, which maintains information on prescriptions from approximately 50,400 retail pharmacies, representing 92% of all prescriptions in the United States. Changes in naloxone dispensing from 2012 to 2018 were examined nationally, by U.S. Census region, and by county urban/rural status (i.e., metropolitan, micropolitan, and rural) (*10*). Annual dispensing rates were calculated by dividing the number of naloxone prescriptions by U.S. Census population estimates per 100,000 persons. CDC analyzed naloxone dispensing in 2018 by age group, sex, out-of-pocket costs, and method of payment.

To assess naloxone dispensing relative to high-dose opioid dispensing, CDC calculated the number of naloxone prescriptions dispensed per 100 high-dose (≥50 MME per day) opioid prescriptions, overall, and by prescriber specialty, U.S. Census region, and urban/rural status in 2017 and 2018, as well as the number of prescriptions and unique patients to whom naloxone and high-dose opioids were dispensed.

CDC also examined naloxone prescriptions at the county level from 2,881 (91.7%) U.S. counties in 2018. Multivariable logistic regression models were fit to identify county-level factors associated with being a high-dispensing (top quartile per 100,000 population) and low-dispensing (bottom quartile) county. The following county-level characteristics were obtained from the American Community Survey: percentage male, non-Hispanic white, disabled, and without a high school diploma, insurance status, unemployment rate, and poverty rate. Urban/rural status* was obtained from CDC’s National Center for Health Statistics. High-dose opioid dispensing rates were calculated; drug overdose death rates were obtained from the National Vital Statistics System. Potential buprenorphine treatment capacity was calculated using data from the Substance Abuse and Mental Health Services Administration by determining the maximum number of patients who could be treated by providers with buprenorphine-prescribing waivers per 1,000 residents. Analyses were conducted using Stata (version 14.2; StataCorp).

## Results

Naloxone dispensing from retail pharmacies increased substantially from 2012 to 2018, from 1,282 prescriptions (0.4 per 100,000) in 2012 to 556,847 (170.2) in 2018 (Table 1). Substantial increases occurred across all U.S. Census regions and urban/rural categories. In 2018, dispensing rates were highest among micropolitan counties (206.3 per 100,000) and in the South (195.0) and lowest in rural counties (147.4) and in the Midwest (139.9).

**TABLE 1:** Estimated annual number of naloxone prescriptions dispensed and rate* of naloxone dispensing from retail pharmacies — United States, 2012–2018

Characteristic	No. of prescriptions (rate)						
	2012	2013	2014	2015	2016	2017^†^	2018^†^
**All**	**1,282 (0.4)**	**1,597 (0.5)**	**6,588 (2.1)**	**26,231 (8.2)**	**134,109 (41.5)**	**270,710 (83.3)**	**556,847 (170.2)**
**County urbanization level^§^**							
Metropolitan	938 (0.4)	1,237 (0.5)	5,944 (2.2)	22,953 (8.3)	119,005 (42.9)	230,514 (82.4)	472,848 (169.1)
Micropolitan	223 (0.8)	255 (0.9)	416 (1.5)	2,630 (9.7)	11,466 (42.1)	27,893 (102.3)	56,247 (206.3)
Rural	121 (0.6)	105 (0.6)	227 (1.2)	647 (3.4)	3,637 (19.3)	12,303 (65.4)	27,752 (147.4)
**U.S. Census region** ^¶^							
Northeast	165 (0.3)	276 (0.5)	1,568 (2.8)	7,052 (12.6)	32,032 (57.1)	53,259 (95.0)	96,773 (172.5)
Midwest	359 (0.5)	359 (0.5)	1,099 (1.6)	2,949 (4.3)	14,984 (22.0)	39,902 (58.5)	95,555 (139.9)
South	456 (0.4)	361 (0.3)	2,376 (2.0)	11,384 (9.4)	58,307 (47.6)	128,117 (103.7)	243,277 (195.0)
West	302 (0.4)	602 (0.8)	1,545 (2.1)	4,846 (6.4)	28,786 (37.6)	49,432 (63.9)	121,243 (155.5)

**Source:** IQVIA Xponent 2012–2018; data were extracted in 2019. The data reflect approximately 92% of all prescriptions from retail pharmacies and are projected nationally.

* Per 100,000 population.

^†^ Starting with 2017 data, IQVIA changed the frame of measurement from number of prescriptions “dispensed to bin” to number of prescriptions “sold to the patient.” To do this, IQVIA eliminated the effects of voided and reversed prescriptions (prescriptions that were never received by the patient), resulting in a downward shift in naloxone prescriptions dispensed of 19.5% for 2017 and 18.9% for 2018.

^§^ 2013 National Center for Health Statistics Urban-Rural Classification Scheme for Counties was used for the creation of the county type variables. https://www.cdc.gov/nchs/data_access/urban_rural.htm. The three classification levels for counties were 1) metropolitan: part of a metropolitan statistical area; 2) micropolitan: part of a micropolitan statistical area (has an urban cluster of ≥10,000 but <50,000 population); and 3) noncore (i.e., rural): not part of a metropolitan or micropolitan statistical area.

^¶^ *Northeast:* Connecticut, Maine, Massachusetts, New Hampshire, New Jersey, New York, Pennsylvania, Rhode Island, and Vermont. *Midwest:* Illinois, Indiana, Iowa, Kansas, Michigan, Minnesota, Missouri, Nebraska, North Dakota, Ohio, South Dakota, and Wisconsin. *South:* Alabama, Arkansas, Delaware, District of Columbia, Florida, Georgia, Kentucky, Louisiana, Maryland, Mississippi, North Carolina, Oklahoma, South Carolina, Tennessee, Texas, Virginia, and West Virginia. *West:* Alaska, Arizona, California, Colorado, Hawaii, Idaho, Montana, Nevada, New Mexico, Oregon, Utah, Washington, and Wyoming.

In 2018, naloxone dispensing rates were higher for female recipients (187.7 per 100,000) than for male recipients (151.6) and higher for persons aged 60–64 years (362.8) than for any other age group (Supplementary Figure 1, https://stacks.cdc.gov/view/cdc/79933). In 2018, the largest percentage of dispensed naloxone prescriptions were to persons with commercial insurance (51.1%), followed by Medicare (35.9%), Medicaid (10.7%), and self-pay (2.4%). Overall, 42.3% of prescriptions did not require out-of-pocket costs; among the remainder, 24.5% required out-of-pocket costs of <$10.00, 21.9% required out-of-pocket costs of $10.01–$50.00, and 5.8% required out-of-pocket costs >$50.00. Among prescriptions paid for by Medicare, 71.1% required out-of-pocket costs; among prescriptions paid for by Medicaid, 43.8% required out-of-pocket costs; among prescriptions paid for by commercial insurance, 41.5% required out-of-pocket costs; 31.0% of self-pay prescriptions had out-of-pocket costs >$50.00 (Supplementary Figure 2, https://stacks.cdc.gov/view/cdc/79934).

From 2017 to 2018, the number of high-dose opioid prescriptions decreased 21%, from 48.6 million to 38.4 million, and the number of naloxone prescriptions increased 106%, from 270,710 to 556,847 (Table 2). In 2018, an estimated 9 million patients were dispensed a high-dose opioid prescription, and 406,203 were dispensed naloxone. The rate of naloxone prescriptions per 100 high-dose opioid prescriptions increased 150% from 2017 (0.6) to 2018 (1.5), varying widely by prescriber specialty. In 2018, among specialty groups with the most high-dose opioid prescriptions, the rate of naloxone prescriptions per 100 high-dose opioid prescriptions was lowest among surgeons (0.2), pain medicine physicians (1.3), physician assistants (1.3), primary care physicians (1.5), and nurse practitioners (2.3). Among all specialty groups, psychiatrists had the highest rate of naloxone prescriptions dispensed for every 100 high-dose opioid prescriptions (12.9), followed by addiction medicine specialists (12.2) and pediatricians (10.4).

**TABLE 2:** High-dose opioid* and naloxone prescriptions dispensed by prescriber specialty, county urbanization level, and U.S. Census region — United States, 2017–2018

Characteristic	2017			2018					
	High-dose opioid prescriptions	Naloxone prescriptions	Naloxone prescriptions per 100 high-dose opioid prescriptions	High-dose opioid prescriptions	% Change from 2017	Naloxone prescriptions	% Change from 2017	Naloxone prescriptions per 100 high-dose opioid prescriptions	% Change from 2017
	No. (%)	No. (%)		No. (%)		No. (%)			
**All**	**48,607,464 (100.00)**	**270,710 (100.00)**	**0.60**	**38,399,208 (100.00)**	**−21**	**556,847 (100.00)**	**106**	**1.45**	**150**
**Prescriber specialty**									
Primary care^†^	11,361,552 (29.03)	63,336 (29.32)	0.56	9,032,155 (29.45)	−21	133,612 (29.58)	111	1.48	150
Pain medicine^§^	7,113,086 (18.17)	40,192 (18.61)	0.57	5,995,058 (19.54)	−16	76,751 (16.99)	91	1.28	117
Surgery	6,356,264 (16.24)	3,072 (1.42)	0.05	4,415,915 (14.40)	−31	8,252 (1.83)	169	0.19	300
Nurse practitioner	4,104,420 (10.49)	43,189 (20.00)	1.05	3,606,936 (11.76)	−12	83,941 (18.58)	94	2.33	109
Physician assistant	3,813,215 (9.74)	22,408 (10.38)	0.59	3,063,470 (9.99)	−20	39,282 (8.70)	75	1.28	117
Other^¶^	1,984,141 (5.07)	9,878 (4.57)	0.50	1,637,893 (5.34)	−17	28,749 (6.36)	191	1.76	260
Medical subspecialties**	1,079,412 (2.76)	5,821 (2.70)	0.54	843,779 (2.75)	−22	20,646 (4.57)	255	2.45	380
Dentistry**^††^**	1,252,860 (3.20)	270 (0.13)	0.02	739,038 (2.41)	−41	549 (0.12)	103	0.07	400
Obstetrics/Gynecology	848,538 (2.17)	4,014 (1.86)	0.47	554,218 (1.81)	−35	17,286 (3.83)	331	3.12	520
Emergency medicine	920,683 (2.35)	8,656 (4.01)	0.94	544,236 (1.77)	−41	15,312 (3.38)	77	2.81	211
Pediatrics	176,639 (0.45)	6,068 (2.81)	3.44	144,933 (0.47)	−18	15,056 (3.33)	148	10.39	206
Psychiatry	109,084 (0.28)	7,986 (3.70)	7.32	81,274 (0.26)	−25	10,487 (2.32)	31	12.90	77
Addiction medicine	17,632 (0.05)	1,090 (0.50)	6.18	14,826 (0.05)	−16	1,810 (0.40)	66	12.21	97
**County urbanization level** ^§§^									
Metropolitan	40,506,108 (83.33)	230,514 (85.15)	0.57	31,922,158 (83.13)	−21	472,848 (84.92)	105	1.48	150
Micropolitan	5,230,850 (10.76)	27,893 (10.30)	0.53	4,156,759 (10.83)	−21	56,247 (10.10)	102	1.35	180
Rural	2,870,505 (5.91)	12,303 (4.54)	0.43	2,320,289 (6.04)	−19	27,752 (4.98)	126	1.20	200
**U.S. Census region** ^¶¶^									
Northeast	7,595,881 (15.63)	53,259 (19.67)	0.70	6,088,692 (15.86)	−20	96,773 (17.38)	82	1.59	129
Midwest	9,489,742 (19.52)	39,902 (14.74)	0.42	7,219,882 (18.80)	−24	95,555 (17.16)	139	1.32	225
South	20,627,124 (42.44)	128,117 (47.33)	0.62	16,528,879 (43.04)	−20	243,277 (43.69)	90	1.47	150
West	10,894,718 (22.41)	49,432 (18.26)	0.45	8,561,754 (22.30)	−21	121,243 (21.77)	145	1.42	180

**Sources:** IQVIA Real World Data Longitudinal Prescriptions (LRx) 2017–2018 (prescriber specialty); data were extracted in 2019. IQVIA Xponent 2017–2018 (county urbanization level and U.S. Census region); data were extracted in 2019. The data reflect approximately 92% of all prescriptions from retail pharmacies. Data from Xponent are projected nationally. Number of prescriptions by specialty does not sum to the total because the data are not projected.

* High-dose opioid prescriptions are defined as ≥50 morphine milligram equivalents per day.

^†^ Primary care includes family practice, general practice, and internal medicine.

^§^ Pain medicine includes anesthesiology, pain medicine, and physical medicine and rehabilitation.

^¶^ Other includes clinical pharmacology, dermatology, dermatopathology, genetics, hospice and palliative medicine, medical microbiology, naturopathic doctor, neurology, neurophysiology, nuclear medicine, nutrition, occupational medicine, optometry, otology, pathology, pharmacist, podiatry, psychology, radiology, sports medicine, unspecified, and other. Pharmacists are included among other specialties given their limited ability to prescribe opioids.

** Medical subspecialties include allergy, cardiology, cardiovascular, diabetes, endocrinology, gastroenterology, hematology, hepatology, hospitalist, immunology, infectious disease, nephrology, oncology, pulmonary disease, and rheumatology.

^††^ Includes dentists, endodontics, orthodontics, pedodontics, periodontics, and prosthodontics. Oral and maxillofacial surgery are classified as surgery.

^§§^ 2013 National Center for Health Statistics Urban-Rural Classification Scheme for Counties was used for the creation of the county type variables. https://www.cdc.gov/nchs/data_access/urban_rural.htm. The three classification levels for counties were 1) metropolitan: part of a metropolitan statistical area; 2) micropolitan: part of a micropolitan statistical area (has an urban cluster of ≥10,000 but <50,000 population); and 3) noncore (i.e., rural): not part of a metropolitan or micropolitan statistical area.

^¶¶^ *Northeast:* Connecticut, Maine, Massachusetts, New Hampshire, New Jersey, New York, Pennsylvania, Rhode Island, and Vermont. *Midwest:* Illinois, Indiana, Iowa, Kansas, Michigan, Minnesota, Missouri, Nebraska, North Dakota, Ohio, South Dakota, and Wisconsin. *South:* Alabama, Arkansas, Delaware, District of Columbia, Florida, Georgia, Kentucky, Louisiana, Maryland, Mississippi, North Carolina, Oklahoma, South Carolina, Tennessee, Texas, Virginia, and West Virginia. *West:* Alaska, Arizona, California, Colorado, Hawaii, Idaho, Montana, Nevada, New Mexico, Oregon, Utah, Washington, and Wyoming.

Across U.S. counties, the rate of naloxone prescriptions dispensed varied substantially, from an average of 16.2 per 100,000 population in the lowest quartile to 410.0 in the highest quartile (Figure). The rate of naloxone prescriptions per 100 high-dose opioid prescriptions also varied across counties, from an average of 0.2 in the lowest quartile to 2.9 in the highest quartile (Figure). In 2018, the rate of naloxone prescriptions per 100 high-dose opioid prescriptions ranged from 1.5 in metropolitan counties and 1.6 in the Northeast to 1.2 in rural counties and 1.3 in the Midwest; the largest increase in 2018 was in the Midwest (Table 2). In 2018, 236 counties (8.3% of counties with available data), dispensed high-dose opioid prescriptions but did not dispense any naloxone prescriptions.

**FIGURE:**
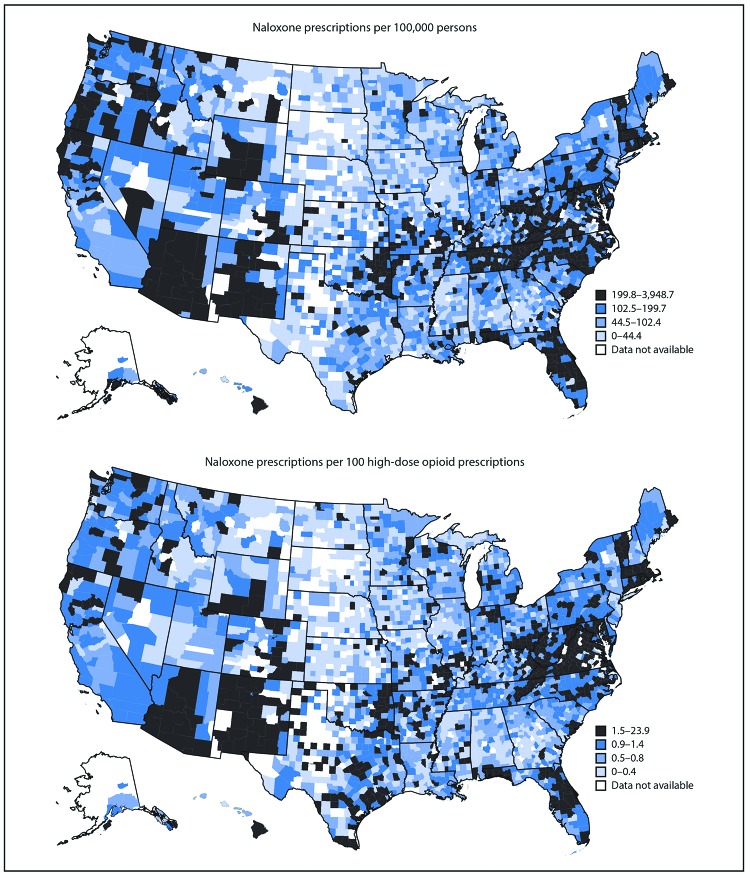
Naloxone prescriptions, by county — United States, 2018

After adjusting for all county characteristics in the multivariable logistic regression models, high naloxone-dispensing counties had higher high-dose opioid dispensing rates, higher drug overdose deaths rates, higher potential buprenorphine treatment capacity, lower percentages of non-Hispanic white residents, higher disability prevalence, and higher rates of Medicaid enrollment (Table 3). Compared with metropolitan counties, micropolitan and rural counties had lower odds of being a high-dispensing county.

**TABLE 3:** County characteristics associated with high- and low-level naloxone dispensing rates — United States, 2018

Characteristic*	High-dispensing counties^†^		Low-dispensing counties^†^	
	OR	p-value	OR	p-value
High-dose opioid dispensing rate (2018)^§^	1.13	<0.001	0.84	<0.001
Drug overdose death rate (2017)	1.02	<0.001	0.97	<0.001
Potential buprenorphine treatment capacity	1.02	0.028	0.95	0.001
Male (%)	0.96	0.175	1.02	0.519
Non-Hispanic white (%)	0.99	0.009	1.01	0.019
Disabled (%)	1.10	<0.001	0.93	0.001
**Insurance status (%)**				
Uninsured	1.01	0.755	1.02	0.304
Medicare	0.99	0.667	1.06	<0.001
Medicaid	1.04	0.004	0.96	0.004
**Unemployment rate**	0.96	0.367	1.01	0.852
**No high school diploma (%)**	0.98	0.157	1.01	0.501
**Income below the Federal Poverty Level (%)**	0.98	0.273	1.03	0.048
**County urbanization level^¶^**				
Metropolitan	Referent	N/A	Referent	N/A
Micropolitan	0.70	0.011	1.12	0.465
Rural	0.46	<0.001	2.61	<0.001

**Abbreviations:** N/A = not applicable; OR = odds ratio.

**Source:** IQVIA Xponent 2018; data were extracted in 2019.

* IQVIA Xponent 2018 (high-dose opioid dispensing rate); American Community Survey (percentage male, percentage non-Hispanic white, percentage disabled, insurance status, unemployment rate, percentage without a high school diploma, poverty rate); National Center for Health Statistics (urban/rural status); National Vital Statistics System (drug overdose death rates); and Substance Abuse and Mental Health Services Administration (potential buprenorphine opioid use disorder treatment capacity). Results are from multivariable logistic regression models that include 2881 U.S. counties.

^†^ According to 2018 naloxone dispensing rates, high-dispensing counties are in the top quartile (199.8–3,948.7 per 100,000), and low-dispensing counties are in the bottom quartile (0–44.4 per 100,000).

^§^ High-dose opioid prescriptions are defined as ≥50 morphine milligram equivalents per day.

^¶^ 2013 National Center for Health Statistics Urban-Rural Classification Scheme for Counties was used for the creation of the county type variables. https://www.cdc.gov/nchs/data_access/urban_rural.htm. The three classification levels for counties were 1) metropolitan: part of a metropolitan statistical area; 2) micropolitan: part of a micropolitan statistical area (has an urban cluster of ≥10,000 but <50,000 population); and 3) noncore (i.e., rural): not part of a metropolitan or micropolitan statistical area.

## Discussion

Naloxone dispensing from retail pharmacies increased substantially from 2012 to 2018. Although naloxone dispensing doubled from 2017 to 2018, dispensing rates remained low, and although high-dose opioid dispensing decreased by 21%, it still remained high. Missed opportunities remain to implement strategies to provide naloxone to patients at risk for overdose. The release of the 2016 CDC Guideline for Prescribing Opioids for Chronic Pain has been associated with accelerated declines in high-dose opioid dispensing (*11*). Additional efforts to implement the guideline recommendations have the potential to improve naloxone dispensing. Nationally, in 2018, only one naloxone prescription was dispensed for every 69 high-dose opioid prescriptions; receipt of a high-dose opioid prescription is a risk factor for overdose. If each provider had considered offering naloxone to every patient receiving a high-dose opioid prescription, as recommended in the CDC guideline, nearly 9 million naloxone prescriptions could have been dispensed, approximately 16 times the 557,000 recorded in 2018. In addition, in one in 12 counties, high-dose opioids were dispensed, but naloxone was not dispensed from a pharmacy. Further, there was a twenty-fivefold variation in naloxone dispensing across counties, with rural counties and the Midwest experiencing the lowest rates despite laws permitting pharmacy-based naloxone dispensing in all 50 states and the District of Columbia (*6*). Naloxone access laws that grant direct authority to pharmacists to dispense naloxone have been associated with reduced fatal opioid overdoses (*12*).

Counties with the greatest need for overdose reversal, (e.g., those with high rates of drug overdose death and high-dose opioid dispensing) tend to have a higher rate of pharmacy-based naloxone dispensing. The highest county-level naloxone dispensing rates were observed in some of the states hit hardest by opioid overdose mortality (e.g., Florida and Massachusetts) and in states that have implemented requirements for naloxone coprescribing (e.g., Arizona and Virginia). Improved access to naloxone holds promise for opioid overdose reversals and the opportunity to link survivors to treatment to prevent a future overdose.

Variation in pharmacy naloxone dispensing rates cannot be fully explained by factors linked to the need for naloxone. Many states have only recently implemented laws requiring coprescription; thus, sufficient time has not passed to examine their full impact. Compared with metropolitan counties, rural counties had a higher likelihood of having low rates of naloxone dispensing, even when controlling for other relevant factors. This is concerning given slower EMS response times and underuse of naloxone by EMS in rural areas relative to the overdose prevalence, which are potentially attributable to resource, certification, and practice constraints (*13*). Harm-reduction programs are more limited in rural areas, and a smaller proportion of rural programs distribute naloxone (*14*). Thus, pharmacy naloxone dispensing holds great promise for positive impact in rural communities.

Clinicians have reported a lack of knowledge and low levels of self-efficacy in counseling patients about overdose and naloxone (*15*). Factors that increase risk for overdose include a history of overdose or substance use disorder, opioid dosages ≥50 MME per day, and concurrent use of benzodiazepines, all of which are indications for prescribing naloxone that providers should consider (*4*). Efforts such as academic detailing, virtual mentoring, and electronic health record alerts can further educate and prompt clinicians about naloxone prescribing (*16*–*18*). Specialties that prescribe higher numbers of high-dose opioids and serve patients at risk for overdose, but were found in the current analysis to have markedly lower rates of naloxone prescriptions dispensed per high-dose opioid prescription (e.g., primary care providers, nurse practitioners, and physician assistants), as well as pain medicine specialists and surgeons, could particularly benefit.

In addition to overcoming prescribing and dispensing barriers, out-of-pocket costs and the rising cost of naloxone present challenges (*19*). Persons without insurance have the highest out-of-pocket costs, with ≥30% of naloxone prescriptions requiring out-of-pocket costs >$50 in 2018. In contrast, approximately one half of prescriptions received by patients with commercial insurance or Medicaid had no out-of-pocket costs, and fewer than one in 10 patients paid >$50. Although naloxone prescriptions among Medicare Part D patients have been increasing, recent research indicates that only a small minority of patients at high risk for overdose in Medicare Part D in 2017 received naloxone (*20*). In this study, patients covered by Medicare paid more, with more than two thirds of prescriptions requiring out-of-pocket costs. In April 2019, the Centers for Medicare and Medicaid Services encouraged Medicare Part D plan sponsors to lower cost-sharing for naloxone (*21*).

The findings in this report are subject to at least five limitations. First, prescriptions reflect those dispensed by pharmacies through either standing orders or clinician prescription; distribution through other channels was not recorded. Second, this analysis was not able to distinguish between prescriptions dispensed under a standing order and those prescribed directly to a patient by a clinician or dispensed to family and friends through third-party authority. Third, available data do not permit assessment of patient factors that might indicate overdose risk and naloxone need; comparing the number of high-dose opioid prescriptions with naloxone prescriptions is an approximation. Fourth, county-level analyses were aggregated by the county where naloxone was dispensed; persons who received these prescriptions and lived in a different county from the pharmacy were not part of the population denominator for the county in which naloxone was dispensed. Finally, the analyses were unable to examine, and findings might not reflect, the impact of recent state policies (e.g., laws requiring coprescription of naloxone).

Comprehensively addressing the opioid overdose epidemic will require efforts to improve naloxone access and distribution in tandem with efforts to prevent initiation of opioid misuse, improve opioid prescribing, implement harm reduction strategies, promote linkage to medications for opioid use disorder treatment, and enhance public health and public safety partnerships. Distribution of naloxone is a critical component of the public health response to the opioid overdose epidemic. Last year, the U.S. Surgeon General called for heightened awareness and availability of naloxone to reverse the effects of opioid overdose, and the U.S. Department of Health and Human Services issued guidance on populations at risk for opioid overdose and thus candidates for naloxone prescribing; pharmacies are a critical venue to help realize expanded access to naloxone (*22*,*23*).

**Summary**

**What is already known about this topic?**

In 2017, 47,600 persons died from drug overdoses involving opioids. Naloxone, a drug that can temporarily reverse the effects of opioids, can help prevent overdose deaths.

**What is added by this report?**

Naloxone dispensing from retail pharmacies increased from 2012 to 2018, with substantial increases in recent years. Despite increases, in 2018, only one naloxone prescription was dispensed for every 69 high-dose opioid prescriptions. The lowest rates of naloxone dispensing were observed in the most rural counties.

**What are the implications for public health practice?**

Additional efforts are needed to improve naloxone access at the local level, including prescribing and pharmacy dispensing. Distribution of naloxone is a critical component of the public health response to the opioid overdose epidemic.

## Footnotes

* 2013 National Center for Health Statistics Urban-Rural Classification Scheme for Counties was used for the creation of the county type variables. https://www.cdc.gov/nchs/data_access/urban_rural.htm. The three classification levels for counties were 1) metropolitan: part of a metropolitan statistical area; 2) micropolitan: part of a micropolitan statistical area (has an urban cluster of ≥10,000 but <50,000 population); and 3) noncore (i.e., rural): not part of a metropolitan or micropolitan statistical area.
